# Molecular epidemiological study on ticks and tick-borne protozoan parasites (Apicomplexa: *Cytauxzoon* and *Hepatozoon* spp.) from wild cats (*Felis silvestris*), Mustelidae and red squirrels (*Sciurus vulgaris*) in central Europe, Hungary

**DOI:** 10.1186/s13071-022-05271-1

**Published:** 2022-05-21

**Authors:** Sándor Hornok, Sándor A. Boldogh, Nóra Takács, Jenő Kontschán, Sándor Szekeres, Endre Sós, Attila D. Sándor, Yuanzhi Wang, Barbara Tuska-Szalay

**Affiliations:** 1 Department of Parasitology and Zoology, University of Veterinary Medicine, Budapest, Hungary; 2 Department of Nature Conservation, Aggtelek National Park Directorate, Jósvafő, Hungary; 3 Plant Protection Institute, Centre for Agricultural Research (ELKH), Budapest, Hungary; 4 Budapest Zoo and Botanical Garden, Budapest, Hungary; 5 Department of Parasitology and Parasitic Diseases, University of Agricultural Sciences and Veterinary Medicine, Cluj-Napoca, Romania; 6 Department of Basic Medicine, School of Medicine, Shihezi University, Shihezi, China

**Keywords:** Cytauxzoon, Hepatozoon, *Ixodes*, *Haemaphysalis*, 18S rRNA gene, Transstadial

## Abstract

**Background:**

Among live wild mammals adapted to urban and peri-urban habitats in Europe, members of the families Felidae, Mustelidae and Sciuridae deserve special attention as pathogen reservoirs because all of these families include members that are kept as pets. We report here the results of our study on two important groups of tick-borne protozoan parasites in ticks and tissues of wild cats, mustelids and red squirrels.

**Methods:**

DNA was extracted from the tissues of carnivores (wild cats, mustelids; *n* = 16) and red squirrels (*n* = 4), as well as from ixodid ticks (*n* = 89) collected from these hosts. These DNA extracts were screened for piroplasms and *Hepatozoon* spp. using conventional PCR analysis and sequencing. In addition, 53 pooled samples of 259 questing *Haemaphysalis concinna* ticks were evaluated for the presence of *Hepatozoon* DNA, followed by phylogenetic analyses.

**Results:**

One wild cat was found to be coinfected with *Cytauxzoon europaeus* and a new genotype of *Hepatozoon felis*, and two additional wild cats were infected with *H. felis* from a different phylogenetic group. In mustelids, *Hepatozoon martis* and two further *Hepatozoon* genotypes were detected. The latter clustered separately, close to others reported from eastern Asia. In addition, *Hepatozoon sciuri* was detected in red squirrels. Morphologic and molecular analyses verified eight tick species. One wild cat was infected with a *H. felis* genotype that was significantly different from that in *Ixodes ricinus* females infesting this cat. Only three pools of questing *H. concinna* nymphs tested positive for *Hepatozoon*, one of which contained *H. martis*.

**Conclusions:**

This study provides the first evidence of the occurrence of any *Cytauxzoon* species and of three *Hepatozoon* species in Hungary. In addition to *H. martis*, two further mustelid-associated *Hepatozoon* genotypes were detected, one of which was new in terms of phylogenetic and broader geographical contexts. This may be the first indication that *H. felis* genotypes from both of its phylogenetic groups occur in Europe. This also appears to be the first evidence of *H. felis* and *C. europaeus* coinfection in felids in Europe, and of autochthonous *H. felis* infection in wild cats north of the Mediterranean Basin. New tick–host associations were also observed in this study. Based on the results, *H. felis* and *H. martis* might survive transstadially in *I. ricinus* and *H. concinna*, respectively.

**Graphical Abstract:**

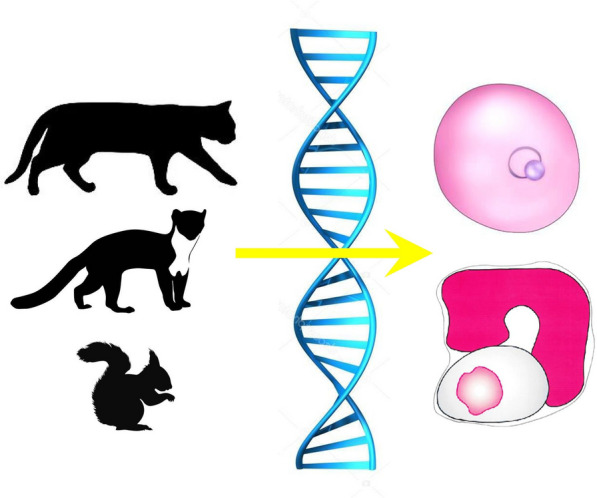

**Supplementary Information:**

The online version contains supplementary material available at 10.1186/s13071-022-05271-1.

## Introduction

Ticks (Acari: Ixodidae) are frequently regarded as the most important transmitters of vector-borne pathogens in Europe [1, 2]. Among tick-borne pathogens, the vast majority of protozoan parasites belong to the phylum Apicomplexa. This phylum includes two categories of (mostly) tick-borne protozoa of major veterinary importance: the genus *Hepatozoon* and three genera of piroplasms (*Babesia*, *Theileria* and *Cytauxzoon*). Tick-borne species in these categories use ticks as biological vectors in different ways. Piroplasms are able to infect vertebrate hosts via tick saliva during a tick-bite. In contrast, *Hepatozoon* species do not reach the salivary gland of ticks during their development and, therefore, infection of a vertebrate host with tick-borne *Hepatozoon* species takes place via oral uptake of infected ticks [3].

In the context of the epidemiology of tick-borne diseases, wild animals are considered to be important reservoirs, from which livestock and pet animals can be infected via ticks shared by these hosts at different stages of the tick life-cycle [4]. This scenario is promoted by the growing interface between urban and natural habitats [5]. From the point of view of mammals that occur in urban/synanthropic environments, with the exception of bats, carnivores and rodents may pose the highest risk of introducing ticks and tick-borne pathogens into human settlements and areas of animal-keeping facilities.

Among European carnivores, members of the family Mustelidae frequently live in urban or peri-urban areas or utilize agricultural landscapes [6]. Their populations are also increasing in several European countries [7, 8]. Importantly, mustelids are frequently kept in captivity as household pets [9] and may become tick-infested when they are walked, increasing the significance of knowledge on their tick-borne pathogens. Another important carnivore that is associated with rural and peri-urban habitats is the European wild cat (*Felis silvestris*), which is the most common wild felid species in Europe [10]. There is evidence that *F. silvestris *populations are hybridizing with domestic cats [10], reflecting interactions between wild and domestic feline hosts and implying that there are opportunities for ticks and tick-borne pathogens to cross between them.

Considering rodents living in parks and gardens in Europe, urban environments can support higher population densities of red squirrels (*Sciurus vulgaris*) than rural areas [11]. At the same time, several countries face the problem of invasive grey squirrels (*Sciurus carolinensis*), against which red squirrel populations should be protected [12]. Thus, knowledge on ticks and tick-borne pathogens affecting red squirrels is an important concern from the point of view of both issues, i.e. urbanization and biodiversity conservation efforts.

In light of the above, it is not surprising that during the past few years increasing attention has been paid to tick-borne protozoan parasites of felids, mustelids and red squirrels in Europe. With respect to piroplasms infecting feline hosts, it has recently been established that three different *Cytauxzoon* species exist in wild and domestic cats on the European continent. Accordingly, *Cytauxzoon europaeus*, *C. banethi* and *C. otrantorum* have been described as new species from samples collected in Germany, Czech Republic, Croatia and Bosnia and Herzegovina [13].

Among the *Hepatozoon* species occurring in wild and domestic cats as typical hosts, *Hepatozoon felis* appears to be the geographically most widespread [14]. In Europe, up to now, autochthonous infections with this species have almost exclusively been restricted to southern countries [15]. *Hepatozoon silvestris*, another* Hepatozoon* species affecting felids, has recently been described in wild cats from Bosnia and Herzegovina [16], and it was subsequently reported in Italy and the UK. This new species was also identified as the cause of a fatal infection in a domestic cat in Switzerland [17]. In addition, a new *Hepatozoon* species, *H. martis*, was discovered in martens (Mustelidae) in Croatia and Bosnia and Herzegovina [18], and also reported from badgers in Spain [19]. Last but not least, although *H. sciuri* is long known to occur in some European countries (e.g. the UK, Italy [20]), its molecular properties have been assessed in a large-scale survey only very recently [21].

Since the above surveys focused on southern Europe and involved eastern, western or central European countries only occasionally, but not Hungary, the present study was initiated to compensate for the current lack of molecular-phylogenetic data on *Hepatozoon* species and piroplasms in tissues of wild cats, mustelids and squirrels in Hungary (also including a canid, the raccoon dog which has not been hitherto analyzed for ticks and tick-borne protozoa in this country and is known to share its habitats with Mustelidae [22]). It was also within the scope of this work to analyze tick infestation of these animals. In addition, *Hepatozoon* DNA was screened in a large set of *Haemaphysalis concinna* ticks collected from the vegetation in 2007 on account of the suspected (or potential) vector role of this and closely related tick species in the transmission of *Hepatozoon* species, as previously proposed based on data from Hungary [23], Austria [18] and Japan [24].

## Methods

For this study, 20 mammals were sampled, including 15 wild carnivores (representatives of six species) and four red squirrels (Table 1) which were found dead between 2015 and 2021, almost exclusively in Aggtelek National Park and its surroundings (northeastern Hungary). In addition, an EDTA-anticoagulated blood sample was collected from a tick-infested wolverine at the Budapest Zoo during regular veterinary care. The fur coat of each sampled animal was carefully checked for the presence of ticks, which were collected into vials containing 96% ethanol and stored until analysis. Tick species were identified according to standard keys [25–27]. In addition, pooled samples of 259 questing *H. concinna* ticks (88 males, 48 females, 52 larvae and 71 nymphs analyzed in 29, 16, 1 and 7 pools, respectively) from a previous, countrywide survey [28] were also evaluated for *Hepatozoon* DNA.

**Table 1:** Data of sample collection and results of molecular analyses of carnivores and on-host ticks

Order	Family	Host species	Year of collection	Region in Hungary	PCR status of hosts for *Hepatozoon*/piroplasms	Result of sequencing	*18S* gene GenBank accession numbers	Tick species (stage or sex) collected from hosts	*Tick* sp. stage (*n* = PCR positive for *Hepatozoon* [piroplasms]/all)
Carnivora	Felidae	European wild cat (*Felis silvestris silvestris*)	2015	Northeast	+/−	*Hepatozoon felis*	OL960187	*I. kaiseri* (L)	None
			2016	Northeast	−/−	–	–	*I. kaiseri* (2L)	None
			2019	Northeast	+/+	*Hepatozoon felis*/*Cytauxzoon europaeus*	OM422755/OL409181	*I. ricinus* (2F)	*I. ricinus* F: 2/2 (1/2)
			2021	Northeast	+/−	*Hepatozoon felis*	OL960187	–	–
	Mustelidae	Beech marten (*Martes foina*)	2015	Northeast	−/−	–	–	–	–
			2017	Northeast	+/−	*Hepatozoon* sp.	OL960188	–	–
			2021	North	+/−	*Hepatozoon martis*	OL960183	–	–
		European pine marten (*Martes martes*)	2017	Northeast	+/−	*Hepatozoon martis*	OL960183	*I. ricinus* (M, 5F, L)	*I. ricinus* M: 1/1, F: 5/5, L: 1/1
			2019	Northeast	+/−	*Hepatozoon* sp.	OL960184	*I. ricinus* (2N), *H. concinna *(N, 8L), *D. marinatus* (6L), *D. reticulatus* (L)	*I. ricinus* N: 1/2; *H. concinna* N: 0/1, L: 3/8; *D. marginatus* L: 3/6; *D. reticulatus* L: 1/1
			2019	Northeast	+/−	*Hepatozoon martis*	OL960183	*H. concinna* (2L)	None
			2020	South	+/−	*Hepatozoon martis*	OL960183	*I. ricinus* (F)	None
		Least weasel (*Mustela nivalis*)	2019	Northeast	+/−	*Hepatozoon* sp.	OL960185	*I. acuminatus* (3F), *H concinna* (N, 4L)	*I. acuminatus* F: 1/3; *H. concinna* N: 0/1, L: 1/4
			2015	Northeast	−/−	–	–	–	–
		Wolverine (*Gulo gulo*)	2020	North (zoo)	−/−	–	–	*I. hexagonus* (F)	None
		Eurasian otter (*Lutra lutra*)	2021	Northeast	−/−	–	–	*I. canisuga* (3L)	None
	Canidae	Raccoon dog (*Nyctereutes procyonoides*)	2019	Northeast	−/−	–	–	*I. ricinus* (5N, 7L), *H. concinna* (5N, 6L)	None
Rodentia	Sciuridae	Red squirrel (*Sciurus vulgaris*)	2017	Northeast	−/−	–	–	*I. ricinus* (3N, 3L), *D. marginatus* (2N)	None
			2017	Northeast	−/−	–	–	*I. ricinus* (2F, 8N, 3L)	None
			2018	Northeast	+/−	*Hepatozoon sciuri*	OL960186	–	–
			2019	Northeast	+/−	*Hepatozoon sciuri*	OL960186	–	–

The exact locations of collection are shown in Additional file 2: Table S2

*M* male, *F* female, *N* nymph, *L* larva. *I. Ixodes*, *D. Dermacentor*. *H. Haemaphysalis*

### DNA extraction and PCR methods

For DNA extraction from tissues, samples were taken from the middle of organs to exclude surface contamination, using a sterile scalpel blade or scissors. All tissues were kept frozen at − 20 °C until processing. The DNA was extracted individually from 200 μl of collected blood or approximately 10 mg of spleen or 100 mg liver using the QIAamp DNA Mini Kit (Qiagen, Hilden Germany) according to the manufacturer’s tissue protocol, including extraction control (180 μl tissue lysis buffer processed in each group of samples) to monitor cross-contamination. In addition, DNA was extracted from ticks, with the protocol including an overnight digestion at 56 °C in tissue lysis buffer containing proteinase-K.

Primers and cycling conditions of the PCR analyses are summarized in Additional file 1: Table S1. In the conventional PCR used to detect piroplasms (including *Cytauxzoon* spp.), 5 µl of extracted DNA was added to 20 µl of reaction mixture containing 1.0 U HotStar Taq Plus DNA Polymerase (5 U/µl) (Qiagen), 0.5 µl dNTP Mix (10 mM), 0.5 µl of each primer (50 µM), 2.5 µl of 10× Coral Load PCR buffer (15 mM MgCl_2_ included) and 15.8 µl distilled water. In the PCR amplifying a fragment of the* 18S* rRNA gene of *Hepatozoon* spp. (approx. 650 bp), the protocol was modified by using 0.2 µl of each primer (50 µM), 1 µl extra MgCl_2_ (to a total of 25 mM) and 17.9 µl distilled water.

 Sequence-verified positive controls were included in all PCR analyses: (i) in the* 18S* rRNA PCR for piroplasms, *Babesia canis* DNA (dog/In7); (ii) in the* 18S* rRNA PCR for *Hepatozoon* spp., *Hepatozoon canis* DNA (dog/Dima48); (iii) in the *Cytauxzoon*-specific PCRs amplifying part of the cytochrome *c* oxidase subunit I (*cox*1) and cytochrome *b* (*cytb*) genes, *Cytauxzoon* sp. DNA (from this study).

### Sequencing and phylogenetic analyses

Purification and sequencing of the PCR products were performed by Biomi Ltd. (Gödöllő, Hungary). The newly generated sequences were submitted to GenBank under accession numbers OL409181 (for the shorter* 18S* rRNA sequence of *Cytauxzoon*); OL960183-OL960187, OM422754-OM422755 (for the shorter* 18S* rRNA gene sequences of *Hepatozoon* spp.; OM256565, OM283285 and OM421666 (for the longer sequences [*18S* rRNA, *cox*1, *cytb* genes] of *Cytauxzoon*, respectively); OM256566-OM256569 and OM422756 (for the long* 18S* rRNA gene fragment of *Hepatozoon* spp.); OM200350 (for the *cox*1 gene of *Ixodes hexagonus*); and OM200035-OM200066 for the* 16S* rRNA gene sequences of tick species (Table 1; Additional file 2: Table S2).

Sequences were aligned and compared to reference GenBank sequences by nucleotide BLASTn program (https://blast.ncbi.nlm.nih.gov). All sequences retrieved from GenBank and included in the phylogenetic analysis had 98–100% coverage with sequences from this study. In the main phylogenetic analysis, including all *Hepatozoon* spp. and genotypes from this study, only the shorter* 18S* rRNA sequences could be used because for several closely related species/genotypes the longer* 18S* rRNA sequences generated here had no corresponding sequences (with similar length) in GenBank. This dataset was resampled 1000 times to generate bootstrap values. Phylogenetic analysis was conducted by using the maximum-likelihood method, and Tamura 3-parameter or Jukes-Cantor model according to the best-fit selection with the program MEGA 7.0 [29].

## Results

### Molecular and phylogenetic analyses of piroplasms and *Hepatozoon* spp. in host tissues

Only one host individual, a wild cat (*F. silvestris*) was positive in the PCR analysis aimed at detecting piroplasms. Sequencing of the shorter and longer* 18S* rRNA gene fragments revealed the presence of a *Cytauxzoon* sp. in this animal, with 100% (1227/1227 bp) identity to several sequences available in GenBank from domestic cats in Switzerland (MF503141-MF503146, KU306941-KU306948). The long fragment of the *cox*1 gene of this haplotype had 100% (1524/1524 bp) sequence identity with only two GenBank sequences, one (MT916269) from Luxembourg and another (MT916265) from Germany. The species was determined to be *Cytauxzoon europaeus*, based on its 99.9% (1358/1360 bp) *cox*1 and 100% (1220/1220 bp) *cytb* sequence identities with those from the type material of this species (MT916240 and MT916191, respectively).

Twelve samples were positive in the PCR analysis aimed at detecting *Hepatozoon* species. The rate of PCR positivity was 75% (3/4) among wild cats, 66.7% (2/3) among beech martens, 100% (4/4) among European pine martens, 50% (1/2) among least weasels and 50% (2/4) among red squirrels (Table 1). Sequencing of the shorter and, whenever possible, the longer* 18S* rRNA gene fragments indicated the presence of the species described as follows.

**Fig. 1:**
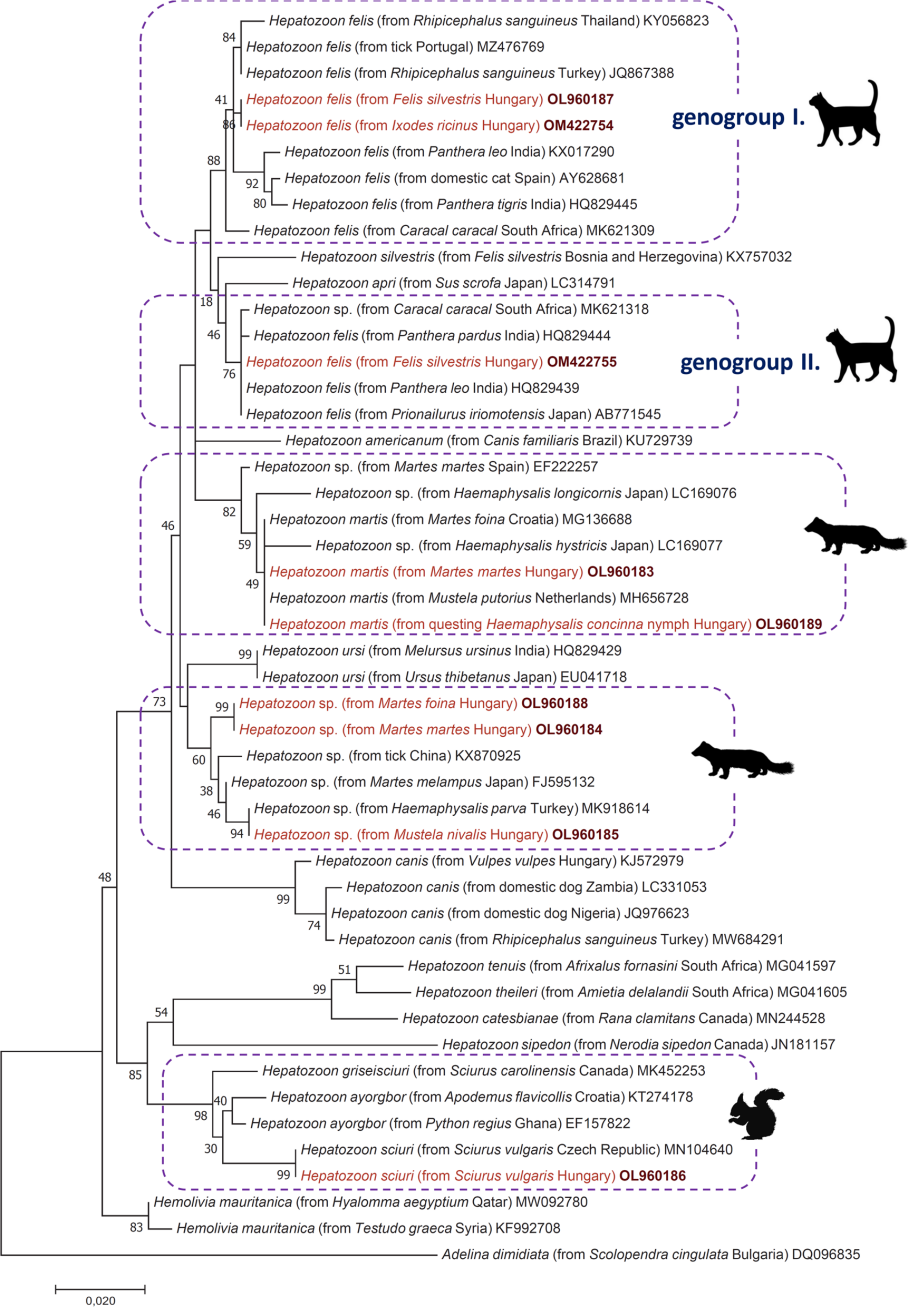
Phylogenetic tree of *Hepatozoon* species and genotypes, constructed with the maximum likelihood method and the Jukes-Cantor model in MEGA 7.0. In each row, after the species or genus name, the isolation source, the country of origin and GenBank accession number are shown. Sequences obtained in this study are indicated in red font and bold accession numbers. The analysis involved 48 nucleotide sequences and 1000 bootstrap replications. There were a total of 586 positions in the final dataset. The scale bar indicates the number of substitutions per site

*Hepatozoon felis* was identified in three wild cats, including the animal with *C. europaeus* infection (Table 1). The more frequent genotype of this species (i.e. detected in 2 wild cats [OL960187] and in 2 *Ixodes ricinus* females [OM422754] collected from the third wild cat) had the highest—but only 99.2% (1627/1640 bp)—sequence identity of its long * 18S* rRNA gene fragment with that of an isolate reported from domestic cat in Spain (AY628681). The phylogenetic tree based on the 650-bp* 18S* rRNA gene fragment showed that this genotype clustered in the clade of “genogroup I”, separately from other isolates of which corresponding sequences were available in GenBank (Fig. 1). Within “genogroup I”, based on the longer * 18S* RNA gene fragment (OM256568), its separation from the clade of the Spanish isolate (AY628681) received high (92%) support (Additional file 3: Figure S1). Importantly, however, from the third wild cat that was coinfected with *C. europaeus*, another *H. felis* genotype was amplified and sequenced (OM422756), showing only 98.2% (1625/1654 bp) * 18S* rRNA sequence identity to that from domestic cat in Spain (AY628681). Phylogenetically, this second *H. felis* genotype clustered in the clade of “genogroup II” (Fig. 1). Based on its longer* 18S* RNA gene fragment, its separation from *H. silvestris* (KX757032) received low (43%) support (Additional file 3: Figure S1).

*Hepatozoon martis* was identified in three European pine martens (*Martes martes*) and in one beech marten (*Martes foina*) (Table 1). All sequences (OM256566, OM256567) were identical with each other, showing 100% (1652/1652 bp) identity in the* 18S* rRNA gene to an isolate reported from Croatia (MG136688). Their phylogenetic clustering confirmed this identity (Fig. 1; Additional file 3: Figure S1).

Two further *Hepatozoon* sp. genotypes were detected in mustelids, which clustered separately from *H. martis* (Fig. 1). One of these (OL960185) originated from a least weasel (*Mustela nivalis*) (Table 1) and had 100% (607/607 bp) sequence identity with two sequences deposited in GenBank: from two tick species (*Haemaphysalis parva* and *Dermacentor marginatus*) collected in Turkey (MK918614 and OM066227, respectively). The second *Hepatozoon* variant detected in both the European pine marten (OL960184) and beech marten (OL960188) in Hungary was a new genotype, showing up to 98.8% (601/608 bp)* 18S* rRNA sequence identity to those from an unreported tick species in China (KX870924- KX870925) and from a Japanese marten (*Martes melampus*) in Japan (FJ595132). The longer* 18S* rRNA sequence of this genotype was successfully amplified and had only 98.4% (1631/1658 bp) identity with that of *Hepatozoon* sp. European pine marten 1 (EF222257). The phylogenetic analysis of this longer sequence revealed separate clustering from both *H. martis* and *Hepatozoon* sp. European pine marten 1, with moderate (89%) bootstrap support (Additional file 3: Figure S1).

In addition, the shorter* 18S* rRNA gene sequence of *H. sciuri* was successfully amplified from two red squirrels (*Sciurus vulgaris*). This genotype had 100% (599/599 bp) sequence identity to several conspecific isolates, reported from the Czech Republic (MN104640), the Netherlands (MH656732) and Spain (EF222259).

It is noteworthy that PCR positivity was not always in agreement between DNA extracts of the spleen and liver from the same host individual. In the great majority of cases, spleen and liver DNA extracts showed parallel positivity or negativity for piroplasms and/or *Hepatozoon* sp. However, from a wild cat and a beech marten only the liver DNA extract tested positive in the PCR analysis for *Hepatozoon* spp., whereas from a least weasel and two red squirrels only the spleen DNA extract had a positive PCR result for *Hepatozoon* spp.

All three carnivore species which were represented by single individuals (Table 1), the wolverine (*Gulo gulo*), the Eurasian otter (*Lutra lutra*) and the common raccoon dog (*Nyctereutes procyonoides*) had PCR-negative results for piroplasms and *Hepatozoon* spp.

### Morphologic and molecular analyses of ticks

Thirteen animals (out of 20) were infested with ticks. Morphologic and molecular analyses of 89 ticks verified the presence of *I. ricinus* and *I. kaiseri* on wild cats; *I. ricinus*, *H. concinna*, *Dermacentor marginatus* and *D. reticulatus* on European pine martens; *I. ricinus* and *I. acuminatus* on least weasels; *I. hexagonus* on wolverine; *I. canisuga* on Eurasian otter; *I. ricinus* and *H. concinna* on raccoon dog, as well as *I. ricinus* and *D. marginatus* on red squirrels (Table 1). None of the beech martens were infested with ticks. In the study material, *I. kaiseri*, *I. canisuga* and *Dermacentor* species were represented only by larvae, *H. concinna* by both larvae and nymphs, *I. hexagonus* and *I. acuminatus* only by females, whereas *I. ricinus* were represented by all developmental stages and both sexes (Table 1).

The *cox*1 or* 16S* rRNA haplotypes of these ticks were either identical or differed by 1–3 bp from specimens already reported in a closer or more distant geographical context, as reflected by GenBank data. This haplotype analysis reflected that while new (not yet reported)* 16S* rRNA sequences of *I. ricinus*, *I. acuminatus* and *D. marginatus* were also obtained, the majority of haplotypes of *Ixodes* species found in this study had already been reported in Europe, whereas those of the two *Dermacentor* species had only been reported in Central Asia (Additional file 2: Table S2). Interestingly, the haplotype of two ticks morphologically identified as *I. ricinus* was 100% identical in its* 16S* rRNA sequence with that of *I. inopinatus* from Algeria (Additional file 2: Table S2).

Molecular analyses of engorged ticks showed that all, some or none of the ticks had PCR-positive results when collected from the same PCR-positive host (Table 1). In the great majority of cases, sequencing yielded the same *Cytauxzoon* or *Hepatozoon* species and genotype in the host tissues and in the tick removed from that host, except for the wild cat coinfected with *C. europaeus* and *H. felis* as described above (Fig. 1). At the same time, ticks from hosts with PCR-negative results always had PCR-negative results (Table 1).

In addition, pooled DNA extracts of 259 questing males, females, nymphs and larvae of *H. concinna* were also tested for the presence of hepatozoon DNA. Only three samples had PCR-positive results, one of which yielded a product that could be sequenced. This sequence showed 100% (1652/1652 bp)* 18S* rRNA gene sequence identity with the *H. martis* isolate demonstrated above from beech marten and European pine marten, as well as with the one reported from Croatia (MG136688). Each of these PCR-positive pools were prepared from *H. concinna* nymphs, including ticks collected in north-northeastern Hungary where the majority of host samples in this study originated.

## Discussion

This study provides the first evidence for the occurrence of any *Cytauxzoon* species and of three *Hepatozoon* species (*H. sciuri*, *H. felis*, *H. martis*) in Hungary, as well as for the occurrence of latter species in central Europe. Two further mustelid-associated *Hepatozoon* genotypes were also detected, one of which was new and closely related to isolates reported so far only in Eastern Asia but not yet in Europe. In addition, the two *H. felis* genotypes found in wild cats in Hungary are new in the phylogenetic context. To the best of our knowledge, this is also the first report of *H. felis* and *C. europaeus* coinfection in any felid in Europe (where this was evaluated, no coinfection was found [30]).

*Cytauxzoon europaeus*, which was identified in a wild cat in the present study, appears to be the most widespread species of its genus in Europe, reported so far in Germany, the Czech Republic, Luxembourg, Bosnia and Herzegovina, Italy, Switzerland and France [14, 31]. While the clinical signs of infection in wild cats, if any, remain to be explored (taking into account that all studies conducted so far used postmortem sampling), domestic cats most often do not show relevant symptoms (e.g. anemia) and remain clinically unaffected [31]. This is in contrast to what is known of *C. felis* in North America [32]. Another major difference in this intercontinental comparison is that while the tick vectors of *C. felis* are known [32], this is not true for any of the European *Cytauxzoon* species. In this study, one of the two *I. ricinus* females that were collected from the *Cytauxzoon*-infected wild cat showed PCR positivity, and sequencing verified the presence of *Cytauxzoon* DNA in this tick. It cannot be ascertained if this is a consequence of taking up piroplasms with the blood meal from the infected cat, or whether this tick may have been the source of infection for its feline host. Nevertheless, as already suggested [33], *I. ricinus* is a likely candidate for a biological vector in the case of *Cytauxzoon* species in Europe.

Among the wild cats included in the present study, the rate of infection was higher with *H. felis* (3 of 4 cats found to be infected) than with *C. europaeus*. Autochthonous infections of domestic cats with *H. felis* have mostly been reported from Mediterranean region of Europe, including Portugal, Spain, France, Italy, Greece and Cyprus [16]. More recently, an isolated case of clinical infection has also been reported in a domestic cat from eastern Austria, the country neighboring western Hungary [34], and imported cases have also been reported in Germany [16]. Nevertheless, to the best of our knowledge, we report here the first case of autochthonous *H. felis* infection in wild cats north of the Mediterranean Basin (previously reported in Bosnia and Herzegovina [10]).

Importantly, the molecular and phylogenetic analyses indicated that two remarkably different genotypes of *H. felis* are present in wild cats in Hungary, clustering within both genogroups “I” and “II”. The first of these (in “genogroup I”) is also remarkably different from other previously reported genotypes of its clade, i.e. from *H. felis* reported from domestic cats (Spain) or Pantherinae (India) and ticks (*Rhipicephalus sanguineus*: Thailand, Turkey). Therefore, the findings of the present study add to the genetic diversity of *H. felis*, as reported previously [15], and warrant further investigations on its taxonomic status and heterogeneity.

The detection of *H. martis* in Hungary in two mustelid species, namely the European pine marten and the beech marten from which it has recently been described (in Bosnia and Herzegovina, as well as in Croatia [18]), is not surprising. However, both of these Balkan countries lie south of Hungary, and thus their mustelid populations are probably well-separated from those in northeastern Hungary sampled in the present study. Since the occurrence of *H. martis* has been reported from south European countries (including Spain [19]) and a sequence is also available in GenBank from Western Europe (MH656728: the Netherlands), the present finding in central Europe adds to the known geographical distribution of this recently described species.

Regarding the two other *Hepatozoon* genotypes detected in three mustelids in Hungary, the *Hepatozoon* sp. from the least weasel showed close molecular and phylogenetic properties to an isolate from host-seeking *H. parva* tick collected in Turkey [35]. In Hungary, *H. parva* has also been reported, but only in the southern part of the country [36]. To the best of our knowledge, this is the first report of this or a similar genotype in Europe. In addition, a new *Hepatozoon* genotype was detected in the known hosts of *H. martis* (i.e. the European pine marten and the beech marten) which showed a significant* 18S* rRNA sequence divergence from *H. martis* and clustered separately from this species. The two genotypes most closely related to this variant were reported in Asia, most importantly in Japanese martens where the infection was associated with granulomatous myocarditis [37]. To the best of our knowledge, this is the first report of this genotype or any other from its phylogenetic group (Fig. 1) in Europe.

In agreement with the overall genetic similarity of *H. sciuri* and the monophyletic clade formed by its sequences from different European countries [21], the* 18S* rRNA gene sequences of this species from Hungary were also identical with each other and with those of this parasite from the Czech Republic, the Netherlands and Spain. It was also reported that PCR-positivity for *H. sciuri* occurred significantly more often for spleen DNA extracts than for liver samples [21]. Consistent with this finding, in both PCR-positive squirrels in our study, only the spleen (but not the liver) was PCR-positive for *H. sciuri*. Although *H. sciuri* is known to infect both of these organs belonging to the haemolymphatic system [20], there still might be differences in the tissue tropism and frequency of *Hepatozoon* developmental stages, and the spleen may harbor more infected white blood cells. In line with this, the rate of positivity was found to be higher in spleen samples than in liver samples among *Cytauxzoon*-infected wild cats [33]. However, in the present study, in a wild cat and a beech marten, the liver DNA extract was PCR-positive for *Hepatozoon* DNA, but not the spleen, highlighting the importance to evaluate both organs simultaneously if possible (and also the myocardium if the expected *Hepatozoon* species has muscle affinity).

The new tick-host associations observed in this study include *Dermacentor* species on European pine marten, *H. concinna* on European pine marten and least weasel and *I. hexagonus* on the wolverine; however, the infestation of most of the mustelid species examined here (and indigenous in Hungary) with *I. ricinus* and of the least weasel with *I. acuminatus* have already been reported [38]. *Ixodes kaiseri* is known to infest wild cats, as reported in the Caucasus region [39] and in the present study, and *I. canisuga* was reported from Eurasian otter in Germany [40], similar to our report in Hungary in the present study. Nevertheless, these are the first records of these tick-host associations in Europe and Hungary, respectively [36]. To the best of our knowledge, the presence of *D. marginatus* is also new on red squirrels, both in Hungary and in a broader geographical context. Since mustelids and squirrels have a comparable preference to use enclosures (burrows or tree holes), they frequently harbor tick species or stages that are endophilic [38, 41], in particular, species of the subgenus *Pholeoixodes* (as exemplified by *I. kaiseri*, *I. hexagonus* and *I. canisuga*), *I. acuminatus* and *Dermacentor* larvae, as supported by findings of the present study.

The results of this study on the presence of protozoan DNA in ticks should be interpreted carefully. It is evident that in the great majority of cases the reason for finding the same *Hepatozoon* species/genotype in a host and its tick(s) is that these were probably ingested with the blood meal, and this scenario does not indicate a vector role. However, the situation is different in the case of the wild cat that was infected with a specific *H. felis* genotype, but from which two engorged female *I. ricinus* ticks were collected with a significantly different *H. felis* genotype. This finding raises the possibility that the latter genotype of *H. felis* may be transmitted transstadially from the nymphal to the adult stage in *I. ricinus*. However, an experimental transmission study is required to ultimately confirm this possibility. The complete life-cycle and the tick vector of *H. felis* are still unknown [34], but this protozoan parasite has already been reported from *I. ricinus* [42]. In line with this finding, during our study, both *I. ricinus* females collected from the infected wild cat contained the DNA of *H. felis*. On the other hand, the *I. kaiseri* larva removed from another infected wild cat was PCR-negative for *H. felis* DNA.

Among the four *H. martis*-infected mustelids only one was infected with ticks. Interestingly, all *I. ricinus* specimens (including 1 male, 5 females and 1 larva) collected from the PCR-positive host contained the DNA of *H. martis*, unlike in the case of any other *Hepatozoon*-infected host in the present study. The *Hepatozoon* genotype closely related to *H. martis* was detected in two tick species collected from its host, the least weasel, i.e. *I. acuminatus* and *H. concinna*, with a low rate of PCR-positivity. On the other hand, the DNA of the *Hepatozoon* genotype more distantly related to *H. martis* was detectable in all four tick species collected from their host, a European pine marten (Table 1).

These data do not specify potential vectors of the detected *Hepatozoon* spp., but they do indicate which tick species infest relevant hosts and have access to *Hepatozoon* gamonts in the study region, and the biological vectors are probably among them. On the contrary, ticks that are not vectors of *Hepatozoon* species may soon digest protozoan DNA together with the blood meal; therefore, negative PCR test results for all engorged larvae or nymphs of a tick species from a PCR-positive host may indicate that the tick species is not a suitable vector. This is well exemplified by our finding that all 21 ticks collected from the *H. sciuri*-infected squirrels had negative PCR test results. In line with this, as already suggested, the biological vectors of *H. sciuri* are most likely not ticks but other arthropods, probably fleas [43, 44].

Last but not least, the DNA of *H. martis* was demonstrated here retrospectively from a pool of questing *H. concinna* nymphs collected in 2007 [28]. The corresponding sequence was identical with that of *H. martis* from two host species examined in this study in Hungary, suggesting that this tick species is a carrier (possibly a vector) of *H. martis*. This is in line with the proposed role of *Haemaphysalis* ticks in the transmission of certain *Hepatozoon* species [23, 24]. In particular, the possibility that *H. concinna* may play a vector role in the transmission of *H. martis* has already been mentioned in the context of unpublished previous data [10], and this is confirmed by the present results. It is noteworthy that only nymphs were found to be PCR-positive from a large set of various tick stages, indicating that in the case of transstadial transmission characteristic of *Hepatozoon* spp. [3], these may have acquired *H. martis* in the larval stage. This possibility is further supported by observations that typically larvae and nymphs, but not adults, of *H. concinna* use mustelids as hosts (Table 1; [36]). However, ultimately only transmission studies can confirm the biological vector role of *H. concinna* in the epidemiology of *H. martis*.

## Conclusions

This study provides the first evidence of the occurrence of any *Cytauxzoon* species and of three *Hepatozoon* species (*H. sciuri*, *H. felis*, *H. martis*) in Hungary. Two further mustelid-associated *Hepatozoon* genotypes were also detected, one of them new both in phylogenetic and broader geographical contexts. To the best of our knowledge, this is the first indication that *H. felis* genotypes from both of its known phylogenetic groups occur in Europe. This also appears to be the first evidence of *H. felis* and *C. europaeus* coinfection in any felid in Europe, and autochthonous *H. felis*-infection was diagnosed for the first time in wild cats north of the Mediterranean Basin. New tick-host associations observed in this study include *Dermacentor* species on European pine marten, *H. concinna* on European pine marten and least weasel and *I. hexagonus* on the wolverine. Our finding of two significantly different genotypes of *H. felis* in a wild cat and its *I. ricinus* ticks suggests that the latter genotype was not acquired with the tick blood-meal, i.e. it might be able to survive transstadially from the nymphal to the adult stage in this tick species. In addition, the presence of *H. martis* DNA exclusively in questing nymph(s) of *H. concinna* raises the possibility that larvae acquired this protozoan parasite and that nymphs might be able to cause infection of another mustelid host if ingested (e.g. during grooming).

## Supplementary Information

**Additional file 1: Table S1.** Primers and cycle conditions of conventional PCRs used in this study.

**Additional file 2: Table S2.** Location of sample collection and sample codes in the same vertical order as in Table 1. Accession numbers refer to sequences of the* 16S* rRNA gene of Ixodidae in GenBank (except for *Ixodes hexagonus *from *Gulo gulo*, of which part of the *cox*1 gene was sequenced).

**Additional file 3: Figure S1.** Phylogenetic tree made with the maximum likelihood method and Tamura 3-parameter model in MEGA 7.0, based on longer* 18S* rRNA gene sequences of *Hepatozoon* species and genotypes. In each row, after the species or genus name, the isolation source, the country of origin and GenBank accession number are shown. Sequences obtained in this study are indicated by red fonts and bold accession numbers. The analysis involved 13 nucleotide sequences and 1000 bootstrap replications. There were a total of 1636 positions in the final dataset. The scale-bar indicates the number of substitutions per site.

## Data Availability

The sequences obtained and/or analyzed during the current study are deposited in GenBank (18S rRNA gene of *Cytauxzoon* and *Hepatozoon* spp.: OL409181, OL960183-OL960187, OM422754-OM422755, OM256565-OM256569 and OM422756; *cox*1 and *cytb* genes of *Cytauxzoon*: OM283285 and OM421666, respectively; *cox*1 gene of *Ixodes hexagonus*: OM200350 and 16S rRNA gene sequences of tick species: OM200035-OM200066). All other relevant data are included in the manuscript and the references or are available upon request by the corresponding author.

## Abbreviations

*cox*1: Cytochrome *c* oxidase subunit I gene
*cytb*: Cytochrome *b* gene
rRNA: Ribosomal RNA
