# Supplementary material for: Molecular epidemiological study on ticks and tick-borne protozoan parasites (Apicomplexa: Cytauxzoon and Hepatozoon spp.) from wild cats (Felis silvestris), Mustelidae and red squirrels (Sciurus vulgaris) in central Europe, Hungary
Source: Parasit Vectors. 2022 May 21;15:174. doi: 10.1186/s13071-022-05271-1 (PMC9123708; doi:10.1186/s13071-022-05271-1)
Supplement: Supplementary file 1 — Additional file 1: Table S1. Primers and cycle conditions of conventional PCRs used in this study. [file 13071_2022_5271_MOESM1_ESM.pdf]

Supplementary Table 1. Primers and cycle conditions of conventional PCRs used in this study.

Target group	Target gene	Primer name	Primer sequence (5'-3')	≈ Length of amplicon (bp)	Thermocycling profile	Reference
Ixodidae	*cox*1	LCO1490	GGTCAACAAATCATAAAGATATTGG	710	95 °C for 5 min; 40× (94 °C for 40 s; 48 °C for 1 min; 72 °C for 1 min); 72 °C for 10 min	Folmer et al. (1994)
		HCO2198	TAAACTTCAGGGTGACCAAAAAATCA			
Ixodidae	16S rRNA	16S+1	CTGCTCAATGATTTTTTAAATTGCTGTGG	460	95 °C for 5 min; 40× (94 °C for 40 s; 51 °C for 1 min; 72 °C for 1 min); 72 °C for 10 min	Black et al. (1994)
		16S-1	CCGGTCTGAACTCAGATCAAGT			
Piroplasms	18S rRNA	BJ1	GTCTTGTAATTGGAATGATGG	500	95 °C for 10 min; 40× (95 °C for 30 s; 54 °C for 30 s; 72 °C for 40 s); 72 °C for 5 min	Casati et al. (2006)
		BN2	TAGTTTATGGTTAGGACTACG			
Hepatozoon spp.	18S rRNA	HepF	ATACATGAGCAAAATCTCAAC	650	95 °C for 5 min; 35× (95 °C for 40 s; 57 °C for 40 s; 72 °C for 60 s); 72 °C for 7 min	Inokuma et al. (2002)
		HepR	CTTATTATTCCATGCTGCAG			
Hepatozoon spp.	18S rRNA	HAM-1F	GCCAGTAGTCATATGCTTGTC	1700	95 °C for 5 min; 40× (95 °C for 30 s; 58 °C for 30 s; 72 °C for 1,5 min); 72 °C for 5 min	Criado-Fornelio et al. (2006); Hodžić et al. (2017)
		HPF-2R	GACTTCTCCTTCGTCTAAG			
Cytauxzoon spp.	*cox*1*	Th-For2	TGGYTKGCTTATTGGTTTGG	1966	95 °C for 5 min; 40× (94 °C for 20 s; 64 °C for 30 s; 68 °C for 2,5 min); 72 °C for 7 min	Schreeg et al. (2016); Panait et al. (2021)
		Piro_mt_R1	ACTTTGAACACACTGCTCG			
		Th-For2*	TGGYTKGCTTATTGGTTTGG	1656	95 °C for 5 min; 40× (94 °C for 20 s; 60 °C for 30 s; 68 °C for 2 min); 72 °C for 7 min	
		Cytaux_260R*	AATTCCCATCTCGCTATCACTTTC			
Cytauxzoon spp.	*cytb**	Cytaux_cytb_F1	CTTAACCCAACTCACGTACC	1434	95 °C for 5 min; 45× (95 °C for 20 s; 53 °C for 30 s; 68 °C for 1,5 min); 68 °C for 7 min	Schreeg et al. (2013); Panait et al. (2021)
		Cytaux_cytb_R3	GGTTAATCTTTCCTATTCCTTACG			
		Cytaux_cytb_Finn*	ACCTACTAAACCTTATTCAAGCRTT	1333	95 °C for 5 min; 45× (95 °C for 20 s; 55 °C for 30 s; 68 °C for 1,5 min); 68 °C for 7 min	
		Cytaux_cytb_Rinn*	AGACTCTTAGATGYAAACTTCCC			
Cytauxzoon sp.	18S rRNA*	7549F	GTCAGGATCCTGGGTTGATCCTGCCAG	1726	95 °C for 5 min; 40× (94 °C for 30 s; 65 °C for 30 s; 68 °C for 2 min); 72 °C for 7 min	Millán et al. (2007); Gallusová et al. (2016)
		7548R	GACTGAATTCGACTTCTCCTTCCTTTAAG			
		Cyt-SSU_F2*	CATGGATAACCGTGCTAATTG	1335	95 °C for 5 min; 40× (94 °C for 30 s; 53 °C for 45 s; 72 °C for 1,5 min); 72 °C for 5 min	
		Cyt-SSU_R4*	AGGATGAACTCGATGAATGCA			

*nested PCR with inner primers: the second reactions include 2µl template from the first round

References

Black WC, Piesman J. Phylogeny of hard and soft-tick taxa (Acari: Ixodida) based on mitochondrial 16S rDNA sequences. Proc. Nat. Acad. Sci. USA 1994;91:10034–10038.

Casati S, Sager H, Gern L, Piffaretti JC. Presence of potentially pathogenic *Babesia* sp. for human in *Ixodes ricinus* in Switzerland. Ann Agric Environ Med. 2006;13:65–70.

Criado-Fornelio A, Ruas JL, Casado N, Farias NA, Soares MP, Müller G, Brumt JG, Berne ME, Buling-Saraña A, Barba-Carretero JC. New molecular data on mammalian *Hepatozoon* species (Apicomplexa: Adeleorina) from Brazil and Spain. J Parasitol. 2006;92:93–99. doi: 10.1645/GE-464R.1.

Folmer O, Black M, Hoeh W, Lutz R, Vrijenhoek R. DNA primers for amplification of mitochondrial cytochrome C oxidase subunit I from diverse metazoan invertebrates. Mel. Marine. Biol. Biot. 1994;3:294–299.

Gallusová M, Jirsová D, Mihalca AD, Gherman CM, D'Amico G, Qablan MA, Modrý D. 2016. *Cytauxzoon* infections in wild felids from Carpathian-Danubian- Pontic space: further evidence for a different *Cytauxzoon* species in European felids. J. Parasitol. 2016; 102: 377–380. doi.org/10.1645/15-881

Hodžić A, Alić A, Prašović S, Otranto D, Baneth G, Duscher GG. *Hepatozoon silvestris* sp. nov.: morphological and molecular characterization of a new species of *Hepatozoon* (Adeleorina: Hepatozoidae) from the European wild cat (*Felis silvestris silvestris*). Parasitology. 2017;144:650–661. doi: 10.1017/S0031182016002316.

Inokuma H, Okuda M, Ohno K, Shimoda K, Onishi T. Analysis of the 18S rRNA gene sequence of a Hepatozoon detected in two Japanese dogs. Vet Parasitol. 2002;106:265–271. doi: 10.1016/s0304-4017(02)00065-1.

Millán J, Naranjo V, Rodríguez A, De La Lastra JMP, Mangold AJ, De La Fuente J. Prevalence of infection and 18S rRNA gene sequences of *Cytauxzoon* species in Iberian lynx (Lynx pardinus) in Spain. Parasitology 2007, 134: 995–1001. doi:10.1017/S003118200700248X

Panait LC, Mihalca AD, Modrý D, Juránková J, Ionică AM, Deak G, Gherman CM, Heddergott M, Hodžić A, Veronesi F, Reichard M, Zieman EA, Nielsen CK, Jiménez-Ruiz FA, Hrazdilová K. Three new species of *Cytauxzoon* in European wild felids. Vet Parasitol. 2021;290:109344. doi: 10.1016/j.vetpar.2021.109344.

Schreeg ME, Marr HS, Tarigo J, Cohn LA, Levy MG, Birkenheuer AJ. Pharmacogenomics of *Cytauxzoon felis* cytochrome b: implications for atovaquone and azithromycin therapy in domestic cats with cytauxzoonosis. J Clin Microbiol. 2013;51:3066-9. doi: 10.1128/JCM.01407-13.

Schreeg ME, Marr HS, Tarigo JL, Cohn LA, Bird DM, Scholl EH, Levy MG, Wiegmann BM, Birkenheuer AJ. Mitochondrial genome sequences and structures aid in the resolution of Piroplasmida phylogeny. PLoS One. 2016;11:e0165702. doi: 10.1371/journal.pone.0165702.
