# Supplementary material for: Molecular epidemiological study on ticks and tick-borne protozoan parasites (Apicomplexa: Cytauxzoon and Hepatozoon spp.) from wild cats (Felis silvestris), Mustelidae and red squirrels (Sciurus vulgaris) in central Europe, Hungary
Source: Parasit Vectors. 2022 May 21;15:174. doi: 10.1186/s13071-022-05271-1 (PMC9123708; doi:10.1186/s13071-022-05271-1)
Supplement: Supplementary file 2 — Additional file 2: Table S2. Location of sample collection and sample codes in the same vertical order as in Table 1. Accession numbers refer to sequences of the 16S rRNA gene of Ixodidae in GenBank (except for Ixodes hexagonus from Gulo gulo, of which part of the cox1 gene was sequenced). [file 13071_2022_5271_MOESM2_ESM.pdf]

Supplementary Table 2. Location of sample collection and sample codes in the same vertical order as in Table 1. Accession numbers refer to sequences of the 16S rRNA gene of Ixodidae in GenBank (except for *Ixodes hexagonus* from *Gulo gulo*, of which part of the *cox*1 gene was sequenced).

Species	Location of collection	Code of tissue DNA samples	Code of tick DNA samples	Tick species: accession number	GenBank example with 100% identity (difference in bp): country
Felis silvestris	Zemplén Mts.	VM1,VM2=WCA1	WCT1	Ik: OM200035	KY962052: Hungary
Felis silvestris	Golop	WCA3	WCT4-5	Ik: OM200036	KY962054: Hungary
Felis silvestris	Szalonna	WCA2	WCT2-3	Ir: OM200037,<br>OM200041	GU074616: France<br>GU074606: Spain
Felis silvestris	Szendrő	WCA13	-	-	-
Martes foina	Mályi	NY3, NY4=WCA8	-	-	-
Martes foina	Kazincbarcika	nyest1, nyest2	-	-	-
Martes foina	Budapest	WCA15	-	-	-
Martes martes	Abaújszántó	WCA4	WCT25-31	Ir: OM200044,<br>OM200049	GU074606 (1 bp): Spain<br>GU074598: Algeria
Martes martes	Damak	WCA5	WCT6-23	Ir: OM200040,<br>OM200045,<br>Dm: OM200060,<br>OM200061,<br>OM200061,<br>OM200061<br>Dr: OM200064	Ir: GU074606 (1 bp),<br>GU074588: Slovakia<br>Dm: MH668402: Kazakhstan<br>MH668401: Kazakhstan<br>MH668401 (2 bp),<br>MH668402 (2 bp),<br>Dr: MH645514: Spain
Martes martes	Trizs	WCA7	WCT56-57	-	-
Martes martes	Kaposvár	WCA11	WCT66	Ir: OM200047	GU074588: Slovakia
Mustela nivalis	Szuhakálló	WCA9	WCT58-65	Ia: OM200058	MH708166 (3bp): France
Mustela nivalis	Aggtelek	ME5-6, WCA10	-	-	-
Nyctereutes procyonoides	Aggtelek	WCA6	WCT32-47,<br>49-55	Ir: OM200038,<br>OM200042,<br>OM200046,<br>OM200051,<br>OM200052,<br>OM200054,<br>OM200056	GU074616: France<br>GU074606: Spain<br>GU074588: Slovakia<br>GU074598 (1 bp): Algeria<br>GU074589: Slovakia<br>GU074590: Slovakia<br>GU074595: Ireland
Gulo gulo	Budapest Zoo	RZS5, RZS6	RZS1	Ih: OM200350	MT659132: Hungary
Lutra lutra	Bánréve	WCA14	WCT88-90	Ic: OM200059	KY962053: Hungary
Sciurus vulgaris	Aggtelek	WRO2	WCT67-74	Ir: OM200043,<br>OM200048,<br>OM200053,<br>OM200057<br>Dm: OM200065,<br>OM200066	GU074606: Spain<br>GU074588: Slovakia<br>GU074589: Slovakia<br>GU074607: Netherlands<br>Dm: MH668402 (2 bp),<br>MH668401: Kazakhstan
Sciurus vulgaris	Jósvafő	WRO3	WCT75-87	Ir: OM200039,<br>OM200043,<br>OM200050,<br>OM200053,<br>OM200055,<br>OM200057	GU074616: France<br>GU074606: Spain<br>GU074598: Algeria<br>GU074589: Slovakia<br>GU074590: Slovakia<br>GU074607: Netherlands
Sciurus vulgaris	Mád	WRO4	-	-	-
Sciurus vulgaris	Abaújszántó	WRO5	-	-	-

Abbreviations in sample code: WCA= wild carnivore, WRO = wild rodent, WCT = wild carnivore or rodent tick.

Abbreviation of tick species: Ir = *Ixodes ricinus*, Ik = *Ixodes kaiseri*, Ic = *Ixodes canisuga*, Ih = *Ixodes hexagonus*, Ia = *Ixodes acuminatus*, Dm = *Dermacentor marginatus*, Dr = *Dermacentor reticulatus*, Hc = *Haemaphysalis concinna*
