# Supplementary figures and images for: Molecular epidemiological study on ticks and tick-borne protozoan parasites (Apicomplexa: Cytauxzoon and Hepatozoon spp.) from wild cats (Felis silvestris), Mustelidae and red squirrels (Sciurus vulgaris) in central Europe, Hungary
Source: Parasit Vectors. 2022 May 21;15:174. doi: 10.1186/s13071-022-05271-1 (PMC9123708; doi:10.1186/s13071-022-05271-1)

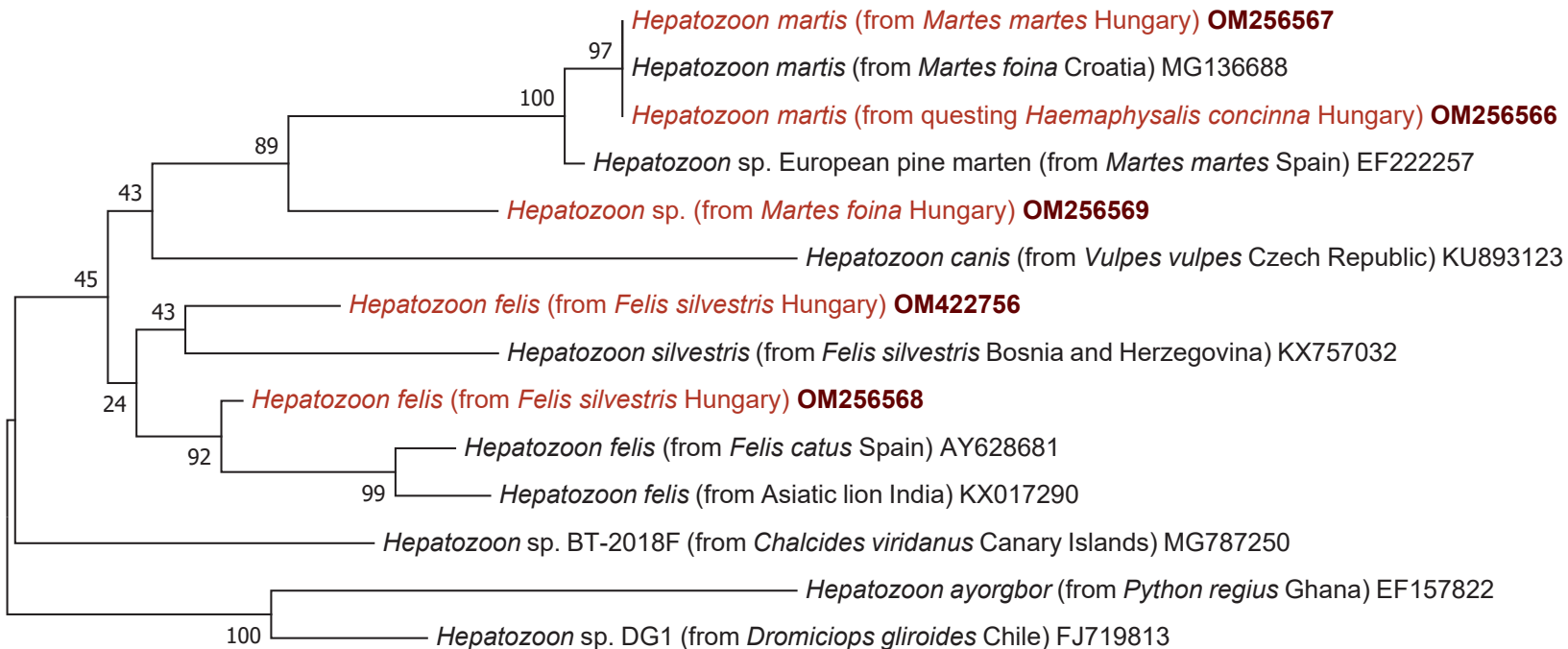

0.0050

Supplement: Supplementary file 3 — Additional file 3: Figure S1. Phylogenetic tree made with the maximum likelihood method and Tamura 3-parameter model in MEGA 7.0, based on longer 18S rRNA gene sequences of Hepatozoon species and genotypes. In each row, after the species or genus name, the isolation source, the country of origin and GenBank accession number are shown. Sequences obtained in this study are indicated by red fonts and bold accession numbers. The analysis involved 13 nucleotide sequences and 1000 bootstrap replications. There were a total of 1636 positions in the final dataset. The scale-bar indicates the number of substitutions per site. [file 13071_2022_5271_MOESM3_ESM.pdf]
